# GPER is involved in the regulation of the estrogen-metabolizing CYP1B1 enzyme in breast cancer

**DOI:** 10.18632/oncotarget.22541

**Published:** 2017-11-20

**Authors:** Francesca Cirillo, Michele Pellegrino, Rocco Malivindi, Vittoria Rago, Silvia Avino, Luigina Muto, Vincenza Dolce, Adele Vivacqua, Damiano Cosimo Rigiracciolo, Paola De Marco, Anna Sebastiani, Sergio Abonante, Miki Nakajima, Rosamaria Lappano, Marcello Maggiolini

**Affiliations:** ^1^ Department of Pharmacy, Health and Nutritional Sciences, University of Calabria, Rende, Italy; ^2^ Regional Hospital Cosenza, Cosenza, Italy; ^3^ Faculty of Pharmaceutical Sciences, Kanazawa University, Kanazawa, Japan

**Keywords:** breast cancer, cancer-associated fibroblasts, CYP1B1, estrogen, GPER

## Abstract

The cytochrome P450 1B1 (CYP1B1) is a heme-thiolate monooxygenase involved in both estrogen biosynthesis and metabolism. For instance, CYP1B1 catalyzes the hydroxylation of E2 leading to the production of 4-hydroxyestradiol that may act as a potent carcinogenic agent. In addition, CYP1B1 is overexpressed in different tumors including breast cancer. In this scenario, it is worth mentioning that CYP1B1 expression is triggered by estrogens through the estrogen receptor (ER)α in breast cancer cells. In the present study, we evaluated whether the G protein estrogen receptor namely GPER may provide an alternate route toward the expression and function of CYP1B1 in ER-negative breast cancer cells, in main players of the tumor microenvironment as cancer associated fibroblasts (CAFs) that were obtained from breast cancer patients, in CAFs derived from a cutaneous metastasis of an invasive mammary ductal carcinoma and in breast tumor xenografts. Our results show that GPER along with the EGFR/ERK/c-Fos transduction pathway can lead to CYP1B1 regulation through the involvement of a half-ERE sequence located within the CYP1B1 promoter region. As a biological counterpart, we found that both GPER and CYP1B1 mediate growth effects *in vitro* and *in vivo*. Altogether, our data suggest that estrogens in ER-negative cell contexts may engage the alternate GPER signaling toward CYP1B1 regulation. Estrogen-CYP1B1 landscape via GPER should be taken into account in setting novel pharmacological approaches targeting breast cancer development.

## INTRODUCTION

Breast cancer is the most frequently diagnosed malignancy and the leading cause of cancer death in women worldwide [1]. A prolonged exposure to estrogens has been considered an important factor driving the initiation and progression of diverse hormone-dependent malignancies, including breast tumor [2]. The multifaceted biological effects triggered by estrogens are mainly mediated by the estrogen receptor (ER)α and ERβ, which acting as ligand-activated transcription factors stimulate cell survival, proliferation and migration [3]. The G protein estrogen receptor, GPER (also known as GPR30), has been recently shown to mediate estrogen action in both normal and malignant cells as well as in main components of the tumor stroma namely cancer-associated fibroblasts (CAFs) [4–5]. In this regard, it has been demonstrated that estrogenic GPER signaling triggers a network of transduction pathways including the transactivation of the epidermal growth factor receptor (EGFR), an increase of intracellular cyclic AMP (cAMP), calcium mobilization, the activation of mitogen-activated protein kinase (MAPK) and phosphatidylinositol 3-kinase/protein kinase B (PI3K/Akt) cascades [6]. These rapid GPER-mediated responses then lead to gene expression changes, cancer cell proliferation and migration [4]. Accordingly, GPER expression has been negatively correlated with relapse free survival and positively associated with tamoxifen resistance in patients with breast tumor [7–8].

Previous studies have indicated that certain metabolites of 17β-estradiol (E2) may influence the development of breast malignancy, therefore great attention has been addressed to a better understanding of the mechanisms involved in estrogen biosynthesis and metabolism as well as in the biological effects of estrogen metabolites [9–10]. For instance, it has been reported that diverse cytochrome P450 enzymes (CYP) contribute to key processes leading to the metabolism of E2 [11]. CYP1B1 (cytochrome P450, family 1, subfamily B, polypeptide 1), which is a heme-thiolate monooxygenase mainly expressed in endocrine-regulated tissues like breast, uterus and ovary, has been indicated as a primary enzyme involved in estrogen metabolism [12]. In addition, CYP1B1 has been suggested to play an essential role in the development of various hormone-dependent tumors, including breast cancer, through the bio-transformation of endogenous estrogens and environmental carcinogens [9, 13–16]. In this context, CYP1B1 is responsible for the metabolism of E2 into 4-hydroxyestradiol (4OHE2) that forms DNA adducts and generates free radicals leading to DNA damage and tumorigenesis in different tissues like breast [2, 17–18]. Several compounds as dioxin, benzo(a) pyrene (BaP) and polycyclic aromatic hydrocarbons (PAHs) stimulate the transcription of CYP1B1 [19–20] as wells as its metabolic activity [2]. It is worth nothing that estrogens generate a feed-forward loop triggering the transcription of CYP1B1, which in turn is primarily involved in the metabolic conversion of these steroids [19, 21–22]. For instance, the transcription of CYP1B1 was induced in breast and endometrial cancer cells by E2 through the activation of ERα and its binding to an estrogen responsive element (ERE) located within the CYP1B1 promoter sequence [21]. These findings may underline the physiological relevance of CYP1B1 regulation by estrogens in the landscape of the estrogen homeostasis and action, in particular in hormone-sensitive tissues [2, 21–22].

In order to provide a more comprehensive scenario through which estrogens may trigger the transcription of CYP1B1 and its metabolic activity in a feed-forward manner, we have ascertained that estrogenic GPER signaling regulates CYP1B1 expression in ER-negative and GPER-positive breast cancer cells, CAFs obtained from breast cancer patients and CAFs derived from a cutaneous metastasis of an invasive mammary ductal carcinoma (met-CAFs). In addition, we have determined that ligand-activated GPER and CYP1B1 contribute to the proliferative responses observed in the aforementioned cells and also in breast tumor xenografts. Thus, GPER may be included among the transduction mediators through which estrogens generate a feed-forward loop driving CYP1B1 expression and its metabolic action toward breast cancer development.

## RESULTS

### E2 and G-1 induce CYP1B1 expression through GPER-mediated signaling

**Figure 1:**
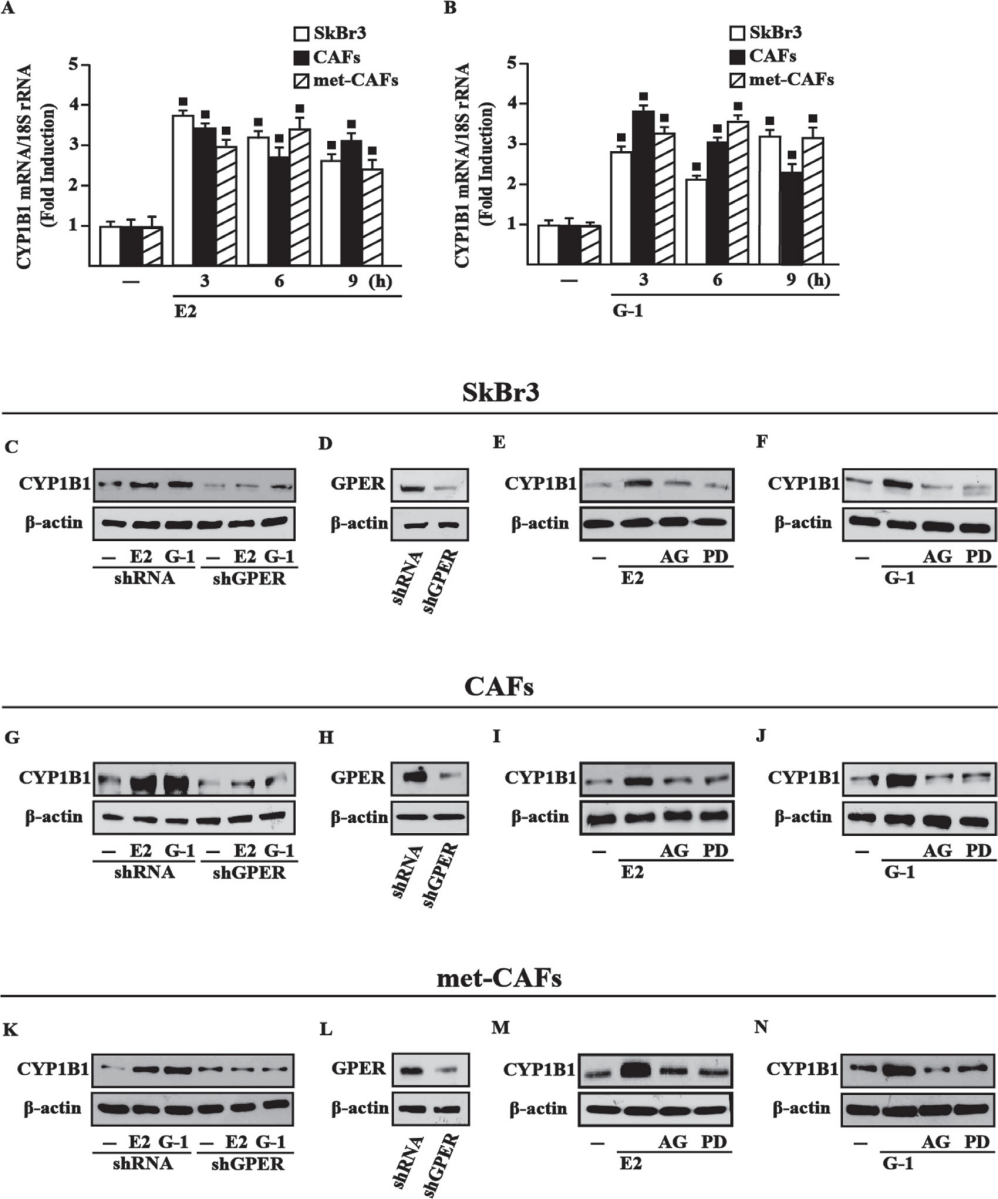
GPER mediates CYP1B1 induction by E2 and G-1 in SkBr3 cells, CAFs and met-CAFs E2 (10 nM) (**A**) and G-1 (100 nM) (**B**) induce the mRNA expression of CYP1B1, as indicated. Data obtained by real-time PCR in three independent experiments performed in triplicate were normalized to 18S expression and shown as fold changes of CYP1B1 expression upon treatments with E2 and G-1 respect to cells treated with vehicle (−). (■) *P* < 0.05 for cells receiving treatments versus vehicle. The up-regulation of CYP1B1 protein levels induced by 10 nM E2 and 100 nM G-1 is abrogated in SkBr3 cells (**C**), CAFs (**G**) and met-CAFs (**K**) transfected for 24 h with shRNA or shGPER and then treated for 6 h with vehicle (−), 10 nM E2 and 100 nM G-1. (**D**, **H**, **L**) Efficacy of GPER silencing. Evaluation of CYP1B1 protein levels in SkBR3 cells (**E**–**F**), CAFs (**I**–**J**) and met-CAFs (**M**–**N**) upon treatment for 6 h with vehicle, 10 nM E2 and 100 nM G-1 alone or in combination with 1 μM EGFR inhibitor AG1478 (AG) or 10 μM MEK inhibitor PD98059 (PD). β-actin serves as a loading control. Results shown are representative of at least two independent experiments.

Previous studies ascertained that estrogens up-regulate CYP1B1 levels through ERα in diverse cancer cells [22], therefore we asked whether estrogens may trigger CYP1B1 expression through GPER in an ER-independent manner. Of note, E2 and the selective GPER agonist G-1 induced CYP1B1 mRNA (Figure 1A–1B) and protein levels (Supplementary Figure 1) in cell contexts lacking ER but expressing GPER as SkBr3 breast cancer cells, CAFs and met-CAFs. Next, the silencing of GPER expression abrogated the CYP1B1 protein induction by E2 and G-1 in SkBr3 cells (Figure 1C–1D), CAFs (Figure 1G–1H) and met-CAFs (Figure 1K–1L). In addition, we found that the EGFR inhibitor AG1478 (AG) and the MEK inhibitor PD98059 (PD) abrogate the increased expression of CYP1B1 upon E2 and G-1 treatments in SkBr3 cells (Figure 1E–1F), CAFs (Figure 1I–1J) and met-CAFs (Figure 1M–1N). Taken together, these findings suggest that the GPER/EGFR/ERK transduction pathway is involved in CYP1B1 expression upon exposure to E2 and G-1 in our model systems.

### A half-ERE site is required for CYP1B1 transcription by E2 and G-1

**Figure 2:**
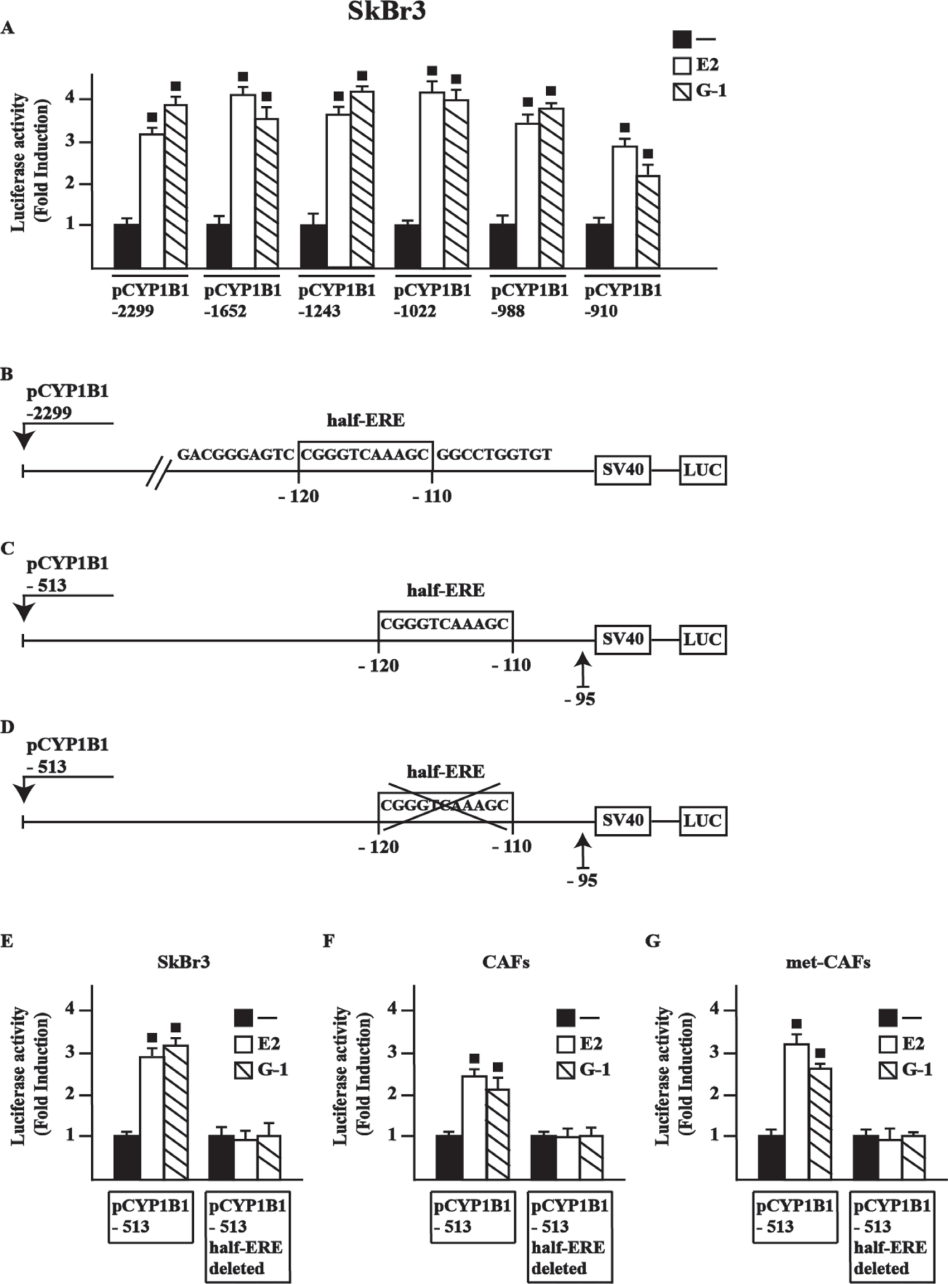
E2 and G-1-stimulate the transcriptional activation of CYP1B1 promoter constructs (**A**) SkBr3 cells were transiently transfected for 8 h with the indicated CYP1B1 promoter constructs, then cells were treated for 18 h with vehicle (−), 10 nM E2 or 100 nM G-1. Schematic representation of the CYP1B1 5′-flanking region containing a half-ERE binding motif (**B**), a deletion construct containing a half-ERE binding motif (**C**) and a deletion construct lacking a half-ERE binding motif (**D**), as indicated. SkBr3 cells (**E**), CAFs (**F**) and met-CAFs (**G**) were transiently transfected for 8 h with the deleted CYP1B1 promoter constructs shown in panels C and D, then treated for 18 h with vehicle, 10 nM E2 and 100 nM G-1, as indicated. The luciferase activities were normalized to the internal transfection control and values of cells receiving vehicle were set as 1-fold induction upon which the activities induced by treatments were calculated. Each column represents the mean ± SD for three independent experiments, each performed in triplicate. (■) indicates *P* < 0.05 for cells receiving treatments versus vehicle.

In order to provide novel insights into the transcriptional activation of CYP1B1 by E2 and G-1, we first ascertained that E2 and G-1 stimulate the luciferase activity of diverse CYP1B1 promoter deletion constructs in SkBr3 cells (Figure 2A), CAFs and met-CAFs (data not shown). Among other sequences, we focused on a half-ERE site [23–24] located from –120 to –110 respect to the transcription initiation site (TIS) of the CYP1B1 promoter (Figure 2B). By site-directed mutagenesis, we generated (see material and methods) two further deleted CYP1B1 promoter constructs containing (Figure 2C) or lacking (Figure 2D) the half-ERE site, respectively. Worthy, E2 and G-1 stimulated the luciferase activity only transfecting in SkBr3 cells (Figure 2E), CAFs (Figure 2F) and met-CAFs (Figure 2G) the plasmid containing the half-ERE site, hence suggesting that this site is involved in CYP1B1 transcription upon treatment with ligands used (see below). Thereafter, the luciferase activity of representative CYP1B1 promoter constructs induced by E2 and G-1 was no longer evident silencing GPER expression or in the presence of the EGFR inhibitor AG1478 (AG) and the MEK inhibitor PD 98059 (PD) in SkBr3 cells (Supplementary Figure 2), CAFs and met-CAFs (data not shown), in accordance with the results shown in Figure 1. Collectively, these findings indicate that E2 and G-1 regulate CYP1B1 transcription through the GPER/EGFR/ERK transduction pathway.

### c-Fos is involved in CYP1B1 expression by E2 and G-1

**Figure 3:**
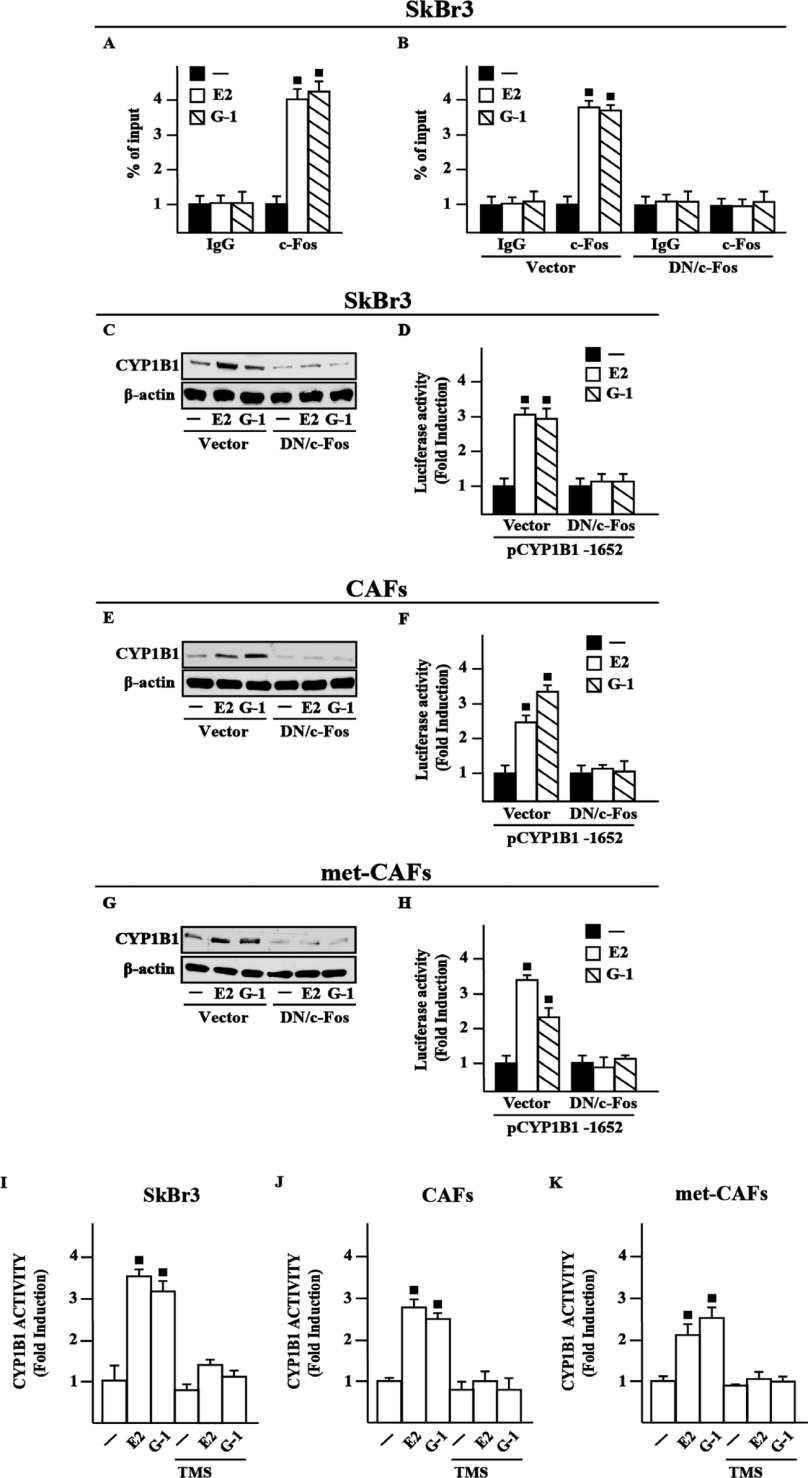
c-Fos is involved in the up-regulation of CYP1B1 by E2 and G-1 in SkBr3 cells, CAFs and met-CAFs (**A**) Recruitment of c-Fos induced by 10 nM E2 and 100 nM G-1 to the half-ERE site located within the CYP1B1 promoter sequence in SkBr3 cells. In control samples non-specific IgG was used instead of the primary antibody. (**B**) SkBr3 cells were transfected for 18 h with a vector or a construct encoding for a dominant negative form of c-Fos (DN/c-Fos), then treated for 3 h with vehicle (−), 10 nM E2 and 100 nM G-1 and thereafter submitted to the chromatin immunoprecipitation procedure using anti-c-Fos or nonspecific anti-IgG antibodies. The amplified sequences were evaluated by real-time PCR. CYP1B1 protein levels in SkBr3 cells (**C**), CAFs (**E**) and met-CAFs (**G**) transfected for 18 h with a vector or DN/c-Fos and then treated for 6 h with vehicle, 10 nM E2 and 100 nM G-1, as indicated. β-actin serves as a loading control. Results shown are representative of at least two independent experiments. SkBr3 cells (**D**), CAFs (**F**) and met- CAFs (**H**) were transfected for 18 h with a CYP1B1construct, a vector or DN/c-Fos and then treated for 18 h with vehicle, 10 nM E2 and 100 nM G-1. The luciferase activities were normalized to the internal transfection control and values of cells receiving vehicle were set as 1-fold induction upon which the activities induced by treatments were calculated. CYP1B1 activity evaluated by EROD assay in SkBr3 cells (**I**), CAFs (**J**) and met-CAFs (**K**) treated for 18 h with vehicle (−), 10 nM E2 and 100 nM G-1 alone or in combination with 5 μM CYP1B1 inhibitor TMS. Fluorescence values of cells receiving vehicle were set as 1-fold induction upon which values induced by treatments were calculated. Each column represents the mean ± SD for three independent experiments, each performed in triplicate. (■) indicates *P* < 0.05 for cells receiving treatments versus vehicle (−).

In order to further assess the transduction mechanisms leading to the CYP1B1 expression, we ascertained that E2 and G-1 trigger c-Fos expression at both mRNA and protein levels in SkBr3 cells (Supplementary Figure 3A–3C), CAFs (Supplementary Figure 3D–3F) and met-CAFs (Supplementary Figure 3G–3I), according to our previous studies [25]. Considering that a half-ERE sequence may differ in only one nucleotide from a canonical AP1 binding site [23–24], we then established that E2 and G-1 trigger the recruitment of c-Fos to the half-ERE site located within the CYP1B1 promoter in SkBr3 cells (Figure 3A), CAFs and met-CAFs (data not shown), however this response was no longer evident transfecting the DN/c-Fos construct in SkBr3 cells (Figure 3B), CAFs and met-CAFs (data not shown). Further supporting these findings, the up-regulation of CYP1B1 protein levels and the transactivation of a representative CYP1B1 construct induced by E2 and G-1 was prevented transfecting SkBr3 cells, CAFs and met-CAFs with the DN/c-Fos (Figure 3C–3H). Taken together, these data indicate that c-Fos is involved in the regulation of CYP1B1 by E2 and G-1.

### CYP1B1 activity is stimulated by E2 and G-1

Previous investigations have suggested that an increased expression of CYP1B1 leads to its enhanced enzymatic activity in cancer cells [14, 26–27]. Therefore, we assessed that a treatment for 18 h with E2 and G-1 stimulates CYP1B1 activity in SkBr3 cells (Figure 3I), CAFs (Figure 3J) and met-CAFs (Figure 3K), as evaluated by EROD assay. Accordingly, we found that the selective CYP1B1 inhibitor named TMS abolishes the CYP1B1 enzymatic activity induced by E2 and G-1 (Figure 3I–3K), thus suggesting its usefulness toward the evaluation of CYP1B1 involvement in certain biological responses (see below).

### GPER and CYP1B1 are involved in the up-regulation of growth regulatory genes by E2 and G-1

**Figure 4:**
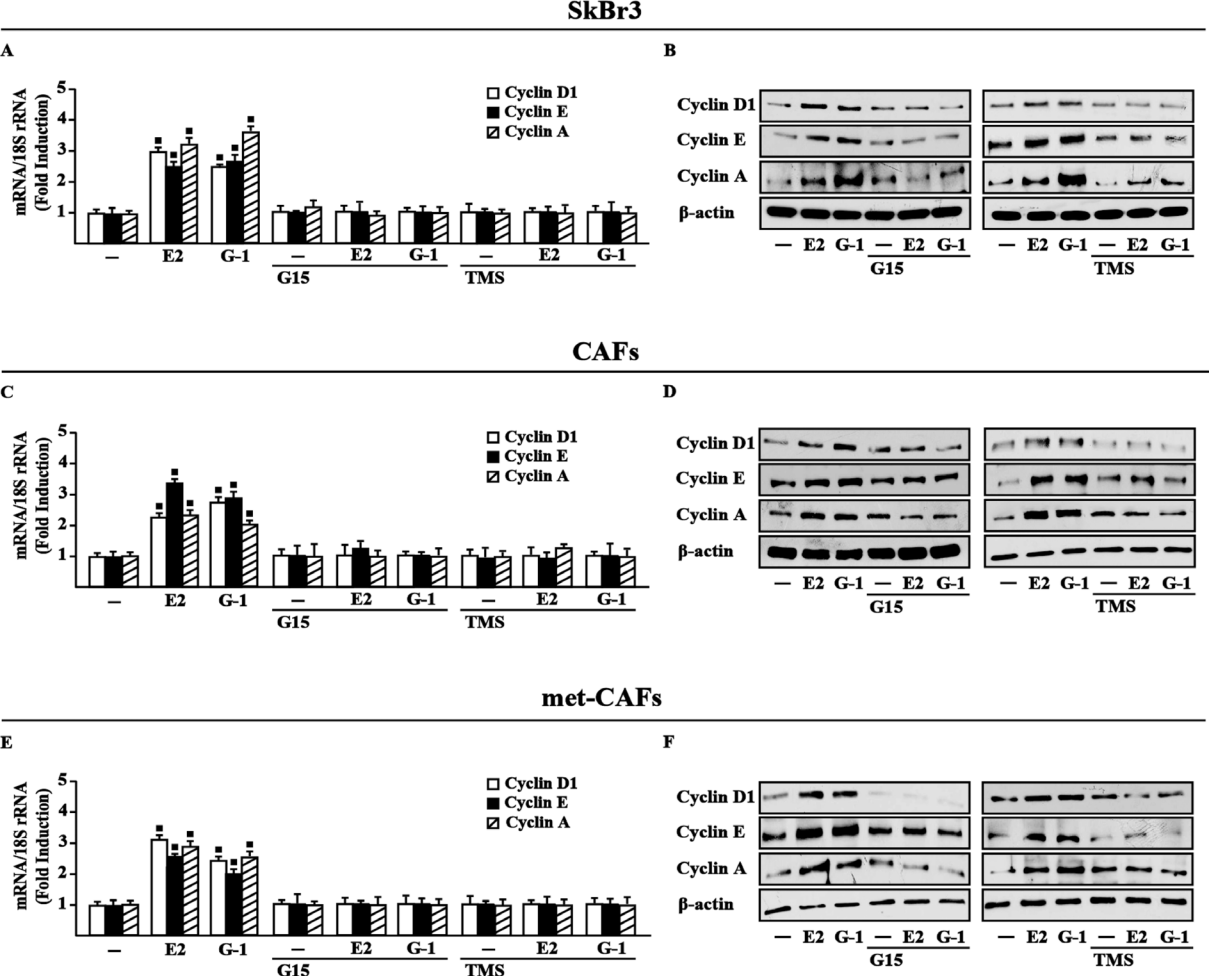
GPER and CYP1B1 mediate the up-regulation of cyclins D1, cyclin E and cyclin A by E2 and G-1 in SkBr3 cells, CAFs and met-CAFs Cyclin D1, cyclin E and cyclin A mRNA expression in SkBr3 cells (**A**), CAFs (**C**) and met-CAFs (**E**) treated for 18 h with vehicle (−), E2 (10 nM) and G-1 (100 nM) alone or in combination with 100 nM GPER antagonist G15 and 5 μM CYP1B1 inhibitor TMS, as evaluated by real-time PCR. Data obtained in three independent experiments performed in triplicate were normalized to 18S expression and shown as fold changes upon E2 and G-1 treatments respect to cells treated with vehicle. (■) *P* < 0.05 for cells receiving treatments versus vehicle. Cyclin D1, cyclin E and cyclin A protein levels in SkBr3 cells (**B**), CAFs (**D**) and met-CAFs (**F**) upon treatments for 18 h with vehicle (−), E2 (10 nM) and G-1 (100 nM) alone or in combination with 100 nM GPER antagonist G15 and 5 μM CYP1B1 inhibitor TMS. β-actin serves as a loading control. Results shown are representative of at least two independent experiments.

**Figure 5:**
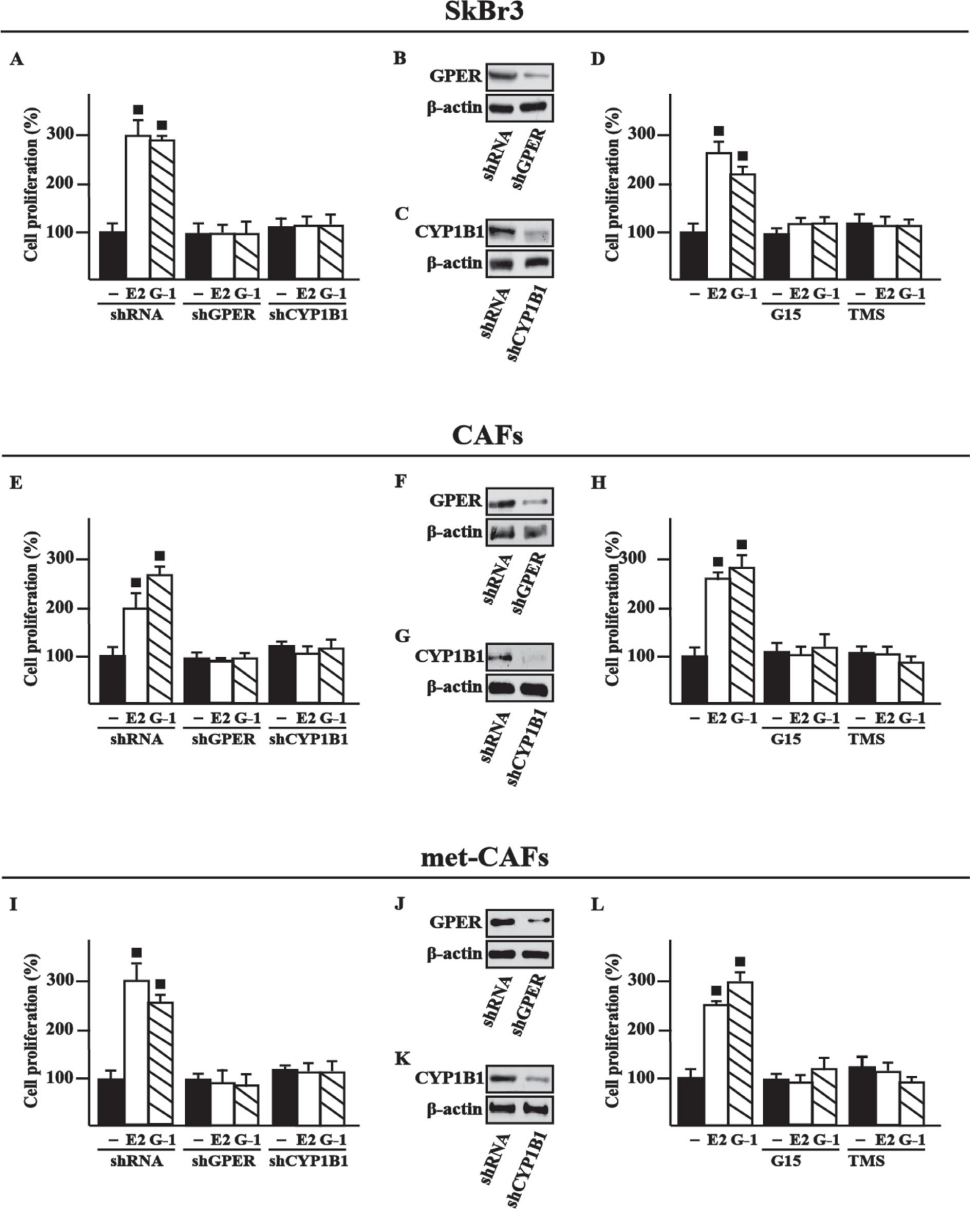
GPER and CYP1B1 are involved in the proliferative effects induced by E2 and G1 in SkBr3 cells, CAFs and met-CAFs The proliferation of SkBr3 cells (**A**), CAFs (**E**) and met-CAFs (**I**) induced by 10 nM E2 or 100 nM G-1 is prevented silencing GPER or CYP1B1 expression. Cells were transfected every 2 days with shRNA, shGPER or shCYP1B1, treated every day with ligands and then counted on day 5. Efficacy of GPER (**B**, **F**, **J**) and CYP1B1 (**C**, **G**, **K**) silencing. β-actin serves as a loading control. The proliferation of SkBr3 cells (**D**), CAFs (**H**) and met-CAFs (**L**) induced by 10 nM E2 or 100 nM G-1 is prevented by 100 nM GPER antagonist G15 and 1 µM CYP1B1 inhibitor TMS. Proliferation of cells treated with vehicle (−) was set as 100% upon which cell growth induced by treatments was calculated. Each data point is the mean ± SD of three independent experiments performed in triplicate. (■) *P* < 0.05 for cells receiving treatments versus vehicle.

Estrogenic GPER signaling has been shown to trigger relevant effects in cancer cells as well as in CAFs through the induction of growth regulators like cyclins [28–30]. Accordingly, we found that E2 and G-1 stimulate the expression of cyclin D1, cyclin E and cyclin A at both mRNA and protein levels in SkBr3 cells, CAFs and met-CAFs, however these responses were abrogated using the GPER antagonist G15 as well as in the presence of the CYP1B1 inhibitor TMS (Figure 4). Nicely fitting with these findings, the proliferative effects elicited by E2 and G-1 in SkBr3 cells, CAFs and met-CAFs were prevented silencing GPER or CYP1B1 as well as in the presence of the GPER and CYP1B1 inhibitors, G15 and TMS, respectively (Figure 5). Taken together, these results suggest that both GPER and CYP1B1 contribute to the growth responses prompted by E2 and G-1 in our model systems.

### GPER and CYP1B1 are involved in the growth effects triggered by E2 and G-1 in breast cancer xenografts

**Figure 6:**
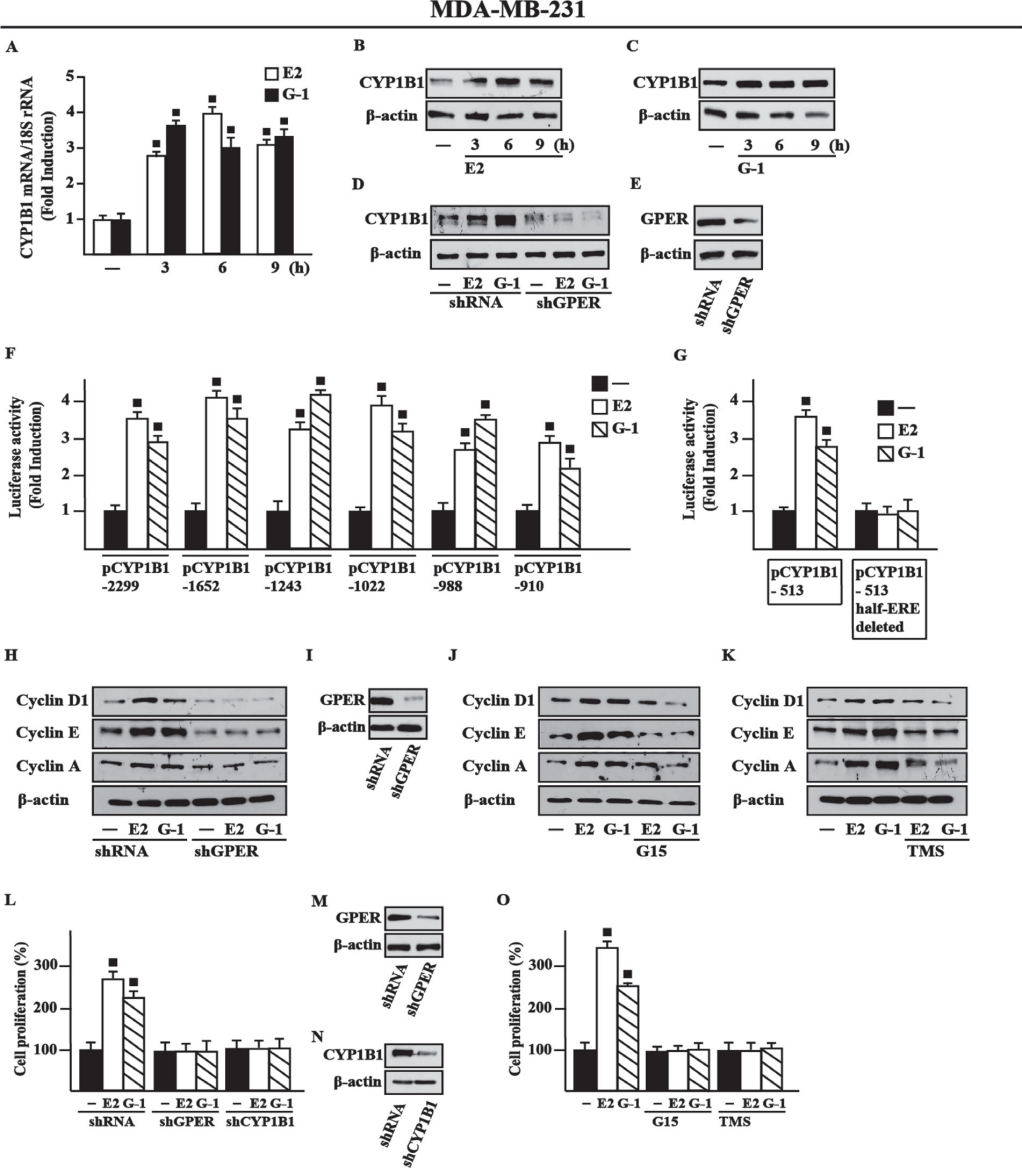
E2 and G-1 induce CYP1B1 expression through GPER in MDA-MB-231 breast cancer cells (**A**) E2 (10 nM) and G-1 (100 nM) induce CYP1B1 mRNA expression in MDA-MB-231 cells, as evaluated by real-time PCR. Data obtained in three independent experiments performed in triplicate were normalized to 18S expression and shown as fold changes upon E2 and G-1 treatments respect to cells exposed to vehicle (−). (**B**–**C**) CYP1B1 protein levels in MDA-MB-231 cells treated with 10 nM E2 and 100 nM G-1, as indicated. (**D**) CYP1B1 protein levels upon treatments with 10 nM E2 and 100 nM G-1 in cells transfected with shRNA or shGPER. (**E**) Efficacy of GPER silencing. β-actin serves as a loading control. Results shown are representative of at least two independent experiments. (**F**) Cells were transiently transfected for 8 h with the indicated CYP1B1 promoter constructs, then cells were treated for 18 h with vehicle (−), 10 nM E2 or 100 nM G-1. (**G**) Cells were transiently transfected for 8 h with the deleted CYP1B1 promoter constructs shown in Figure 2C and 2D, then treated for 18 h with vehicle, 10 nM E2 and 100 nM G-1, as indicated. The luciferase activities were normalized to the internal transfection control and values of cells receiving vehicle were set as 1-fold induction upon which the activities induced by treatments were calculated. Each column represents the mean ± SD for three independent experiments, each performed in triplicate. (**H**) Cyclin D1, cyclin E and cyclin A protein levels in cells transiently transfected with a shRNA or shGPER for 24 h, then treated for 18 h with vehicle, 10 nM E2 or 100 nM G-1. (**I**) Efficacy of GPER silencing. Cyclin D1, cyclin E and cyclin A protein levels in cells treated for 18 h with vehicle, 10 nM E2 and 100 nM G-1 alone or in combination with 100 nM GPER antagonist G15 (**J**) and 5 μM CYP1B1 inhibitor TMS (**K**). β-actin serves as a loading control. Results shown are representative of at least two independent experiments. (**L**) Cell proliferation induced by 10 nM E2 or 100 nM G-1 is prevented silencing GPER or CYP1B1 expression. Cells were transfected every 2 days with shRNA, shGPER or shCYP1B1, treated every day with ligands and then counted on day 5. Efficacy of GPER (**M**) and CYP1B1 (**N**) silencing. β-actin serves as a loading control. (**O**) Cell proliferation induced by 10 nM E2 or 100 nM G-1 is prevented by 100 nM GPER antagonist G15 and 1 µM CYP1B1 inhibitor TMS. Proliferation of cells treated with vehicle was set as 100% upon which cell growth induced by treatments was calculated. Each data point is the mean ± SD of three independent experiments performed in triplicate. (■) *P* < 0.05 for cells receiving treatments versus vehicle.

**Figure 7:**
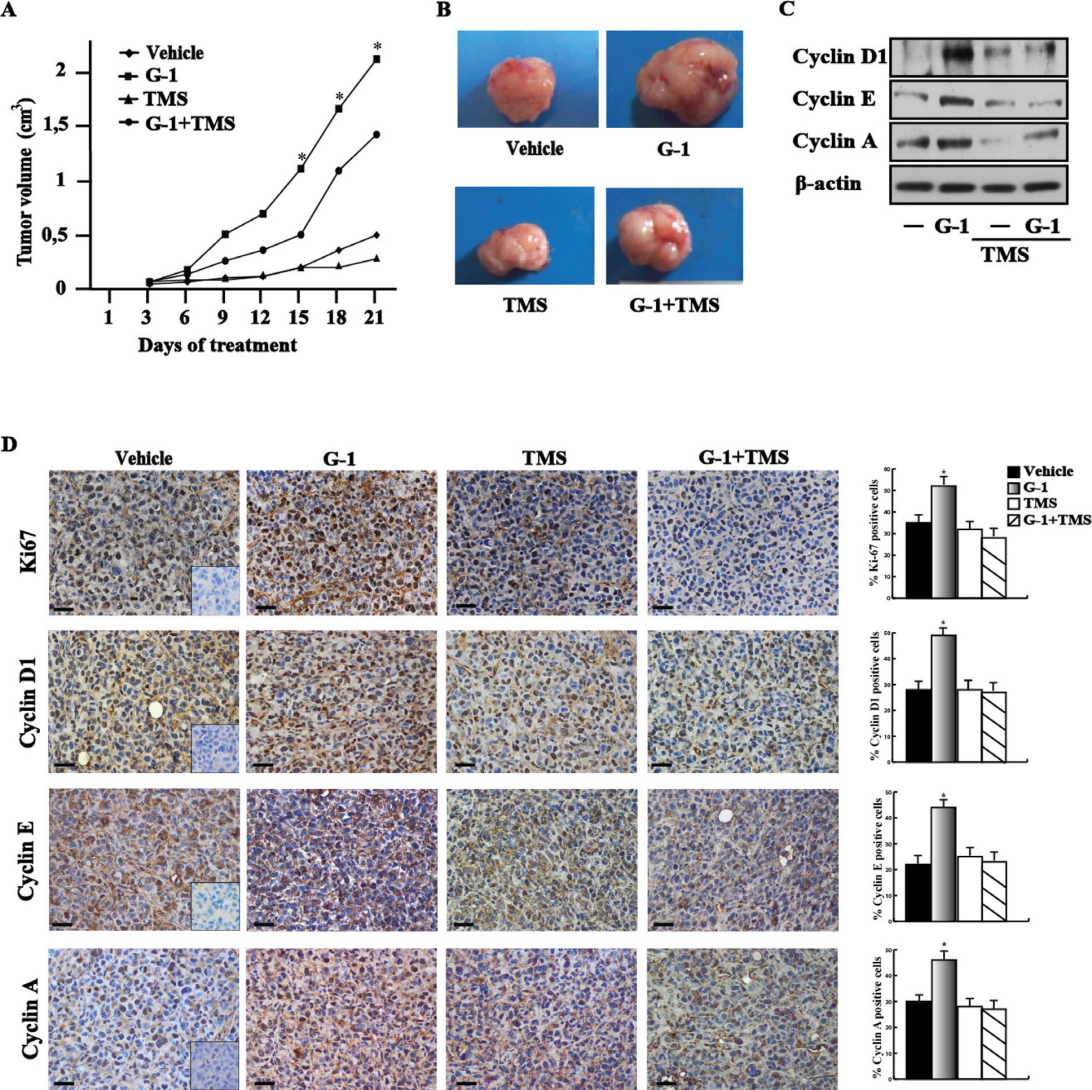
CYP1B1 is involved in the growth of MDA-MB-231 xenografts (**A**) Tumor volume from MDA-MB-231 xenografts implanted in female athymic nude mice treated for 21 days with vehicle, G-1, TMS or both compounds, as indicated. (^*^) indicates *P* < 0.05 for animals treated with G-1 versus animals treated with vehicle. (**B**) Representative images of explanted tumors at day 21, scale bar, 0.3 cm. (**C**) Cyclin D1, cyclin E, cyclin A protein levels in tumor homogenates from MDA-MB-231 xenografts treated as reported above. β-actin serves as loading control. Results shown are representative of two independent experiments. (**D**) Ki67, cyclin D1, cyclin E and cyclin A immunodetection in paraffin embedded sections of explanted tumors from breast cancer xenografts treated with vehicle, G-1 and TMS alone or in combination, as indicated. Scale bar: 25 μm. Insert: negative control. Histograms represent the percentage (± SD) of immunostained positive cells treated with G-1 and TMS alone or in combination versus vehicle treated cells. (^*^) indicates *P* < 0.05.

In order to strengthen the aforementioned observations we turned to the high metastatic and invasive MDA-MB-231 breast cancer cells [31] that we used *in vivo* and *in vitro* studies. First, we determined that E2 and G-1 induce CYP1B1 expression at both mRNA (Figure 6A) and protein levels though GPER (Figure 6B–6E) also in these cells. Corroborating the results obtained in SkBr3 cells, CAFs and met-CAFs, we thereafter ascertained that E2 and G-1 stimulate the luciferase activity of diverse CYP1B1 promoter constructs (Figure 6F) except for the half-ERE deleted plasmid (Figure 6G). Likewise, we found that E2 and G-1 up-regulate the expression of cyclin D1, cyclin E and cyclin A in MDA-MB-231 cells, however these responses were no longer evident silencing GPER (Figure 6H–6I) or using the GPER antagonist G15 (Figure 6J) and the CYP1B1 inhibitor TMS (Figure 6K). Recapitulating the abovementioned findings, E2 and G-1 promoted the proliferation of MDA-MB-231 cells through GPER and CYP1B1, as ascertained silencing their expression (Figure 6L–6N) and using G15 or TMS (Figure 6O). Then, in order to evaluate the role of CYP1B1 in tumor growth *in vivo*, 45-day-old female nude mice were injected with MDA-MB-231 cells into the mammary fat pad region and treated with vehicle, G-1 and TMS alone or in combination. These treatments were well tolerated as no changes in body weight and in food or water consumption were observed together with no evidence of reduced motor function. Among the different groups of mice, no significant difference was assessed after the sacrifice in the mean weights or histologic features of the major organs (liver, lung, spleen and kidney), thus indicating a lack of toxic effects. Of note, TMS treatment prevented the tumor growth induced by G-1 (Figure 7A–7B) and the up-regulation of cyclin protein levels in tumor homogenates (Figure 7C). In addition, an increased expression of the proliferative marker Ki67, together with that of cyclin D1, cyclin E and cyclin A was found in tumor tissue sections obtained from G-1 treated mice with respect to those treated with vehicle (Figure 7D). Worthy, these effects were prevented in the group of animals receiving G-1 in combination with TMS (Figure 7D). Overall, these data suggest that GPER and CYP1B1 are involved in the stimulatory effects exerted by E2 and G-1 in MDA-MB-231 breast cancer cells both *in vitro* and *in vivo*.

## DISCUSSION

**Figure 8:**
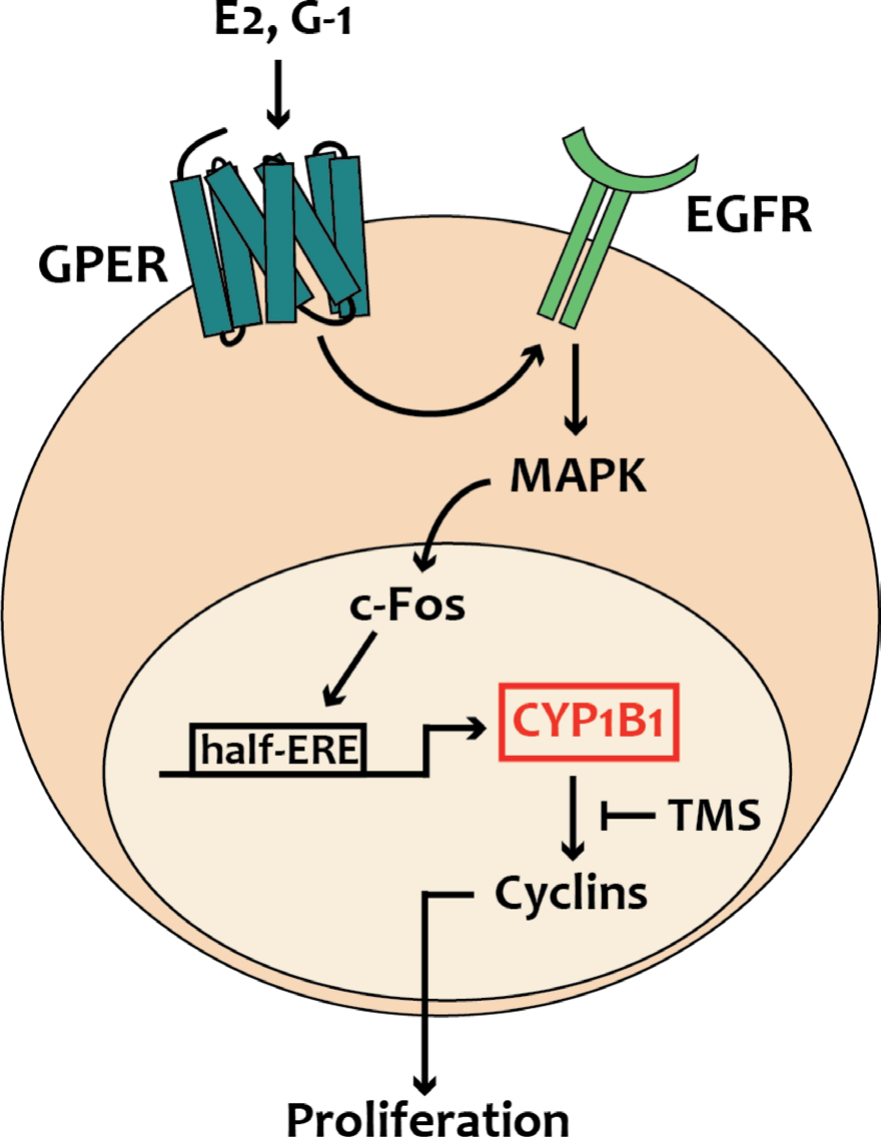
Schematic representation of CYP1B1 regulation by GPER-mediated signaling, as evidenced in breast cancer cells, CAFs and met-CAFs

In the present study we have ascertained that estrogens through the alternate route, namely GPER, regulate CYP1B1 expression and function in diverse ER-negative breast cancer cells, CAFs obtained from breast cancer patients, CAFs derived from a cutaneous metastasis of an invasive mammary ductal carcinoma and in MDA-MB-231 that were used both *in vitro* and *in vivo*. In particular, we have demonstrated that estrogenic GPER signaling stimulates CYP1B1 expression through the activation of the GPER/EGFR/ERK transduction pathway and the recruitment of c-Fos to the half-ERE site located within the CYP1B1 promoter sequence. We have also disclosed that CYP1B1 is involved in the growth effects elicited by GPER ligands, as demonstrated silencing both GPER and CYP1B1 or using their inhibitors named G15 and TMS, respectively. In accordance with these findings, TMS abrogated the increase of the proliferative index Ki67 and the expression of diverse cyclins upon exposure to the selective GPER ligand G-1, as assessed in tumor homogenates and tissue sections. Overall, these findings provide novel evidence regarding the role of GPER on the estrogen-CYP1B1 landscape toward breast tumor progression, as recapitulated in Figure 8.

Estrogens are involved in important physiological functions as the maintenance of the female reproductive system, however these steroids may also contribute to the development of breast malignancies [32]. Estrogen mainly act through the classical ER, nevertheless several studies have demonstrated that GPER can mediate the stimulatory effects of estrogens in both normal and malignant tissues, including breast cancer [4, 28, 33–34]. For instance, ligand-activated GPER triggers a network of transduction pathways such as EGFR, intracellular cyclic AMP, calcium mobilization, MAPK and PI3K, thus leading to the induction of genes involved in the proliferation, migration and invasion of cancer cells including breast tumor cells [33]. Likewise, a clinical correlation between GPER expression and increased tumor size, distant metastasis and recurrence has been found in human breast tumor specimens, suggesting that GPER levels may be predictive of aggressive breast malignancies [7, 34]. Various studies have also revealed that certain GPER-mediated responses to estrogens target important components of the tumor microenvironment driving cancer progression as CAFs [5]. In particular, GPER has been involved in the transcription of genes toward the proliferation, migration and adhesion/spreading of CAFs derived from breast tumor patients [5]. Worthy, in the present study we have ascertained that GPER mediates the stimulatory action of estrogens not only in CAFs obtained from primary breast malignancies but also in CAFs derived from a cutaneous metastasis of an invasive mammary ductal carcinoma. In this regard, it is worthy mentioning that metastasis-associated CAFs may elicit stimulatory effects in metastatic cancer cells similar to those triggered by CAFs at primary tumor sites [35]. Indeed, it is now unquestioned that both tumor growth and the essential steps of the metastatic process are not only dependent on cancer cells, but rather involve a promiscuous interaction between tumor cells and components of the tumor microenvironment as CAFs [36]. Likewise, recent observations have indicated that cancer cells might carry CAFs during their migration to metastatic sites, in such way these co-traveling cells may facilitate tumor development in further tissues [37].

Several studies have suggested that estrogens play a role in the development of hormone-sensitive tumors via oxidative estrogen metabolism [19]. CYP1B1 is a major E2 hydroxylase involved in estrogen biosynthesis and metabolism, generation of DNA damaging pro-carcinogens and resistance to anti-hormone therapies [14]. For instance, CYP1B1 catalyzes the hydroxylation of E2 leading to the formation of 4OHE2 [10], which may trigger the induction of estradiol-3,4-quinone, the strongest ultimate carcinogenic estrogen metabolite that, binding to the N-7 position of guanine, leads to the destabilization of the glycosidic bond and the subsequent DNA depurination and mutagenesis [2, 20, 22, 38]. Considering that CYP1B1 expression has been reported increased in tumor tissues compared to the normal counterpart [16, 39] and given that the levels of 4OHE2 have been found higher in hormone-sensitive tumors like breast cancer respect to normal tissues [20], this cytochrome has attracted increasing interest as potential target in further anticancer strategies, especially in the treatment of hormone-related tumors [40].

The transcription of CYP1B1 is mainly regulated by the aryl hydrocarbon receptor (AhR) that acts as a ligand-activated transcription factor [41]. Xenobiotics like dioxin, halogenated aromatic hydrocarbons, BaP and PAHs, are AhR activators of CYP1B1 transcription [20, 41]. In accordance with our findings, it has been recently reported that G-1 is also able to up-regulate the expression of both AhR and CYP1B1 in ER-positive MCF-7 breast cancer cells, although the molecular mechanisms involved remain to be elucidated [42]. Furthermore, CYP1B1 can be regulated by other transcription factors as AhR nuclear translocator (ARNT) complex (AhR/ARNT), Sp1, cAMP–response element binding protein (CREB) and ER [22]. In this context, it is worth noting that CYP1B1 may be induced by its own substrates [2]. For instance, E2-activated ERα triggered the transcription of CYP1B1 through an estrogen responsive element (ERE) located within the CYP1B1 promoter sequence in MCF-7 cells [21]. These findings may indicate that the regulation of CYP1B1 expression and activity by its own substrates like estrogens would be patho-physiologically important for their metabolism and homeostasis in hormone-responsive tissues. In this scenario, our data provide novel insights into the current knowledge regarding the regulation of CYP1B1 by estrogens. Using as model systems ER-negative breast cancer cells, CAFs from a primary tumor, CAFs from a metastatic site and breast xenografts, we have determined that GPER may be an alternate route toward the regulation of CYP1B1 expression and function by estrogens in different biological targets. Worthy, the CYP1B1 inhibitor TMS prevented the stimulatory effects on tumor growth exerted by estrogenic GPER signaling both *in vitro* and *in vivo*, in accordance with previous studies that highlighted the ability of this agent to delay tumor progression in xenograft models [13]. TMS has been also proposed as a potential chemopreventive agent in hormone-sensitive tumors as it prevented the formation of the carcinogenic estrogen metabolite 4OHE2, it induced apoptotic cell death selectively in cancer cells and it reduced tumor volume of tamoxifen-resistant breast cancer xenografts [11, 13, 15, 43–44]. Collectively, our findings suggest that GPER may be included among the transduction mediators involved by estrogens in the regulation of CYP1B1 toward the development of breast cancer at both primary and metastatic sites.

## MATERIALS AND METHODS

### Reagents

17β-Estradiol (E2), salicylamide (2-hydroxybenzamide), resorufin (7-hydroxy-3H-phenoxazin-3-one) and resorufin ethyl ether (7-ethoxy-3H-phenoxazin-3-one) were purchased from Sigma-Aldrich (Milan, Italy). G-1 (1-[4-(-6-bromobenzol [1,3]diodo-5-yl)-3a,4,5,9b-tetrahidro3H5cyclopenta[c]quinolin-8yl]-ethanone), G-15 (3aS,4R,9bR)-4-(6-bromo-1,3-benzodioxol-5-yl)-3a,4,5,9b-3H-cyclopenta[c]quinolone and TMS 1-[2,(3,5-Dimethoxyphenyl)ethenyl]-2,4-dimethoxybenzene were obtained from Tocris Bioscience (Space, Milan, Italy). Tyrphostin AG1478 (AG) and PD98059 (PD) were obtained from Calbiochem (DBA, Milan, Italy). All the aforementioned compounds were dissolved in dimethyl sulfoxide (DMSO), except for salicylamide that was dissolved in methanol.

### Ethics statement

All procedures conformed to the Helsinki Declaration for the research on humans. Signed informed consent was obtained from all patients and the experimental research has been performed with the ethical approval provided by the “Comitato Etico Regione Calabria, sezione area nord c/o azienda ospedaliera di Cosenza, Italy”.

### Cell cultures

SkBr3 and MDA-MB-231 breast cancer cells were obtained by ATCC (Manassas, VA, USA), used less than 6 months after resuscitation and routinely tested and authenticated according to the ATCC suggestions. SkBr3 cells were maintained in RPMI-1640 (Life Technologies, Milan, Italy) without phenol red, supplemented with 10% fetal bovine serum (FBS) and 100μg/ml penicillin/streptomycin (Life Technologies, Milan, Italy). MDA-MB231 cells were maintained in DMEM (Dulbecco’s modified Eagle’s medium) (Life Technologies, Milan, Italy) with phenol red, with a supplement of 5% FBS and 100 μg/ml of penicillin/streptomycin. CAFs obtained from breast malignancies and met-CAFs obtained from biopsy of cutaneous metastasis in a patient with a primary invasive mammary ductal carcinoma, who previously had undergone surgery, were characterized and maintained as we have previously described [28, 45]. Briefly, specimens were cut into smaller pieces (1–2 mm diameter), placed in digestion solution (400 IU collagenase, 100 IU hyaluronidase, and 10% serum, containing antibiotic and antimycotic solution) and incubated overnight at 37°C. The cells were then separated by differential centrifugation at 90 × g for 2 min. Supernatant containing fibroblasts was centrifuged at 485 × g for 8 min; the pellet obtained was suspended in fibroblasts growth medium (Medium 199 and Ham’s F12 mixed 1:1 and supplemented with 10% FBS) and cultured at 37°C in 5% CO_2_. Primary cells cultures of metastasis-derived fibroblasts were characterized by immunofluorescence. Briefly, cells were incubated with human anti-vimentin (V9) and human anti-cytokeratin 14 (LL001), both from Santa Cruz Biotechnology (DBA, Milan, Italy). To characterize fibroblasts activation, we used anti-fibroblast activated protein α (FAPα) antibody (H-56; Santa Cruz Biotechnology, DBA, Milan, Italy) (data not shown). CAFs and metastasis-derived CAFs were maintained in Medium 199 and Ham’s F12 (mixed 1:1) supplemented with 10% FBS and 100 μg/ml penicillin/streptomycin. All cell lines were grown in a 37°C incubator with 5% CO_2_. All cell lines to be processed for immunoblot and RT-PCR assays were switched to medium without serum and phenol red the day before treatments.

### Gene expression studies

Total RNA was extracted and cDNA was synthesized by reverse transcription as previously described [46]. The expression of selected genes was quantified by real-time PCR using platform Quant Studio7 Flex Real-Time PCR System (Life Technologies). Gene-specific primers were designed using Primer Express version 2.0 software (Applied Biosystems). For CYP1B1, c-Fos, cyclin D1, cyclin E, cyclin A and the ribosomal protein 18 S, which was used as a control gene to obtain normalized values, the primers were: 5′-TGTGCCTGTCACTATTCCTCATG-3′ (CYP1B1 forward) and 5′-GGGAATGTGGTAGCCCAAGA-3′ (CYP1B1 reverse); 5′-CGAGCCCTTTGATGACTTCCT-3′ (c-Fos forward) and 5′-GGAGCGGGCTGTCTCAGA-3′ (c-Fos reverse); 5′-GTCTGTGCATTTCTGGTTGCA-3′ (cyclin D1 forward) and 5′-GCTGGAAACATGCCGGTTA-3′ (cyclin D1 reverse); 5′-GCATGTCACCGTTCCTCCTTG-3′ (cyclin A forward) and 5′-GGGCATCTTCACGCTCTATTTT-3′ (cyclin A reverse); 5′-GATGACCGGGTTTACCCAAAC-3′ (cyclin E forward) and 5′-GAGCCTCTGGATGGTGCAA-3′ (cyclin E reverse); 5′-GGCGTCCCCCAACTTCTTA-3′ (18S forward) and 5′-GGGCATCACAGACCTGTTATT-3′ (18S reverse). Assays were performed in triplicate and the results were normalized for 18 S expression and then calculated as fold induction of RNA expression.

### Western blot analysis

Cells were grown in 10-cm dishes, exposed to treatments and then lysed in 500 μL of 50 mmol/L NaCl, 1.5 mmol/L MgCl2, 1 mmol/L EGTA, 10% glycerol, 1% Triton X-100, 1% sodium dodecyl sulfate (SDS), and a mixture of protease inhibitors containing 1 mmol/L aprotinin, 20 mmol/L phenylmethylsulfonyl fluoride and 200 mmol/L sodium orthovanadate. Protein lysates from tumor homogenates obtained from nude mice were processed as previously described [47]. Protein concentration was determined using Bradford reagent according to the manufacturer’s recommendations (Sigma-Aldrich, Milan, Italy). Equal amounts of whole protein extract were resolved on a 10% SDS-polyacrylamide gel, transferred to a nitrocellulose membrane (Amersham Biosciences, Sigma-Adrich, Milan, Italy), probed overnight at 4°C with antibodies against CYP1B1 (TA339934), cyclin D1 (TA801655), cyclin E (TA590076), cyclin A (TA890057) (OriGene Technologies, DBA, Milan, Italy), c-Fos (E8), GPER (N-15) and β-actin (C-2) (Santa Cruz Biotechnology, DBA). Proteins were detected by horseradish peroxidase-linked secondary antibodies (Santa Cruz Biotechnology, DBA) and then revealed using the chemiluminescent substrate for western blotting Westar Nova 2.0 (Cyanagen, Biogenerica, Catania, Italy).

### Gene silencing experiments

Cells were plated into 10 cm dishes and transfected using X-treme GENE 9 DNA Transfection Reagent (Roche Diagnostics, Sigma-Adrich, Milan, Italy) for 24 h before treatments with a control shRNA, a shRNA for GPER (shGPER) or a shRNA for CYP1B1 (shCYP1B1, Santa Cruz Biotechnology, DBA, Milan, Italy). The silencing of GPER expression was obtained by using the constructs which we have previously described, and used [48].

### Bioinformatic tools

The putative promoter sequences of CYP1B1 was retrieved from the National Center for Biotechnology Information (NCBI) (http://www.ncbi.nlm.nih.gov). Prediction of transcription factors for CYP1B1 was performed using TRANSFAC (http://www.generegulation.com) site.

### Plasmids

The plasmid DN/c-Fos, which encodes a c-Fos mutant that heterodimerizes with c-Fos dimerization partners but does not allow DNA binding, was a kind gift from Dr C Vinson (NIH, Bethesda, MD, USA). pGL3-promoter plasmid containing the 5’-flanking region from –2299 to +25 respect to the transcription initiation site (TIS) [49] of the CYP1B1 gene and CYP1B1 promoter deletion constructs containing fragments –1652 to +25, –1243 to +25, –1022 to +25, –988 to +25, –910 to +25 respect to TIS were generated as previous described [50].

### Site-directed mutagenesis

The p-GL3-promoter plasmid containing the 5′-flanking region from –1652 to +25 respect to TIS of the CYP1B1 gene was used as template to generate as previously described [51] the DNA fragment from –513 to –95 respect to TIS containing a half-ERE site (see results section), which was amplified by PCR using the following primers: sense 5′-CGAGGTACCCTGATCTCGCCGCAAGAACT-3′ and anti-sense 5′-GTCGCTAGCGCCGCACACCAGGCC-3′. The CYP1B1 deletion construct from –513 to –95 lacking the half-ERE site (see results section) was amplified by PCR using the following primers: sense 5′-CGAGGTACCCTGATCTCGCCGCAAGAACT-3′ and anti-sense 5′-GTCGCTAGCGCCGCACACCAGGCCGACTCCCGTCCAGG-3′. The amplified DNA fragments were digested with KpnI and NheI and cloned into the pGL3-promoter plasmid (Promega, Milan, Italy). The sequence of each construct was verified by nucleotide sequence analysis.

### Transfections and luciferase assays

Cells (1 × 10^5^) were plated into 24-well dishes with 500 µl/well of regular growth medium the day before transfection. Growth medium was replaced with medium lacking serum on the day of transfection, which was performed using X-tremeGene9 reagent, as recommended by the manufacturer (Roche Diagnostics), with a mixture containing 0.5 μg of each reporter plasmid and 1 ng of pRL-TK. After 8 h, the medium was replaced with fresh medium lacking serum and the cells were incubated for 18 h with treatments. Luciferase activity was then measured with the Dual Luciferase Kit (Promega, Milan, Italy) according to the manufacturer’s recommendations. Firefly luciferase activity was normalized to the internal transfection control provided by the Renilla luciferase activity. The normalized relative light unit values obtained from cells treated with vehicle (−) were defined as one fold induction, relative to which the activity induced by treatments was calculated.

### Chromatin immunoprecipitation (CHIP) assay

The cells grown on 10-cm plates were shifted for 24 h in a medium lacking serum and then exposed to treatments for 3 h. Thereafter, cells were cross-linked with 1% formaldehyde and sonicated. Supernatants were immuno-cleared with salmon DNA/protein A-agarose (Upstate Biotechnology, Inc., Lake Placid, NY, USA) and immunoprecipitated with anti c-Fos (H-125) or nonspecific IgG (Santa Cruz Biotechnology, DBA). Pellets were washed, eluted with a buffer consisting of 1% SDS and 0.1 mol/L NaHCO3 and digested with proteinase K. DNA was obtained by phenol/chloroform extractions and precipitated with ethanol. A 4 µl volume of each immunoprecipitated DNA sample and input were used as a template to amplify by PCR the region containing a half-ERE site located in the CYP1B1 promoter region. The primers used to amplify this fragment were as follows: 5′-CTGCTGGTAGAGCTCCGAGG-3′ (forward) and 5′-CCCGCTGCTCTGCTTCTTAC-3′ (reverse). Data were normalized to the input for the immunoprecipitation.

### Ethoxyresorufin-O-deethylase activity assay

The cells (7 × 10^4^ cells/ml) were grown in 24-well plates for 48 h, then were shifted for 24 h in a medium lacking serum and then treated for 18 h. The cells were washed with PBS, and fresh medium containing salicylamide to inhibit conjugating enzymes (1.5 mM) was added to the wells. The plate was incubated at 37°C for 5 min, then 7-ethoxyresorufin was added (final concentration of 5 μM) and the reaction was carried out for 1 hour at 37°C with gentle stirring of the plate every 5 min. Aliquots of cell suspensions (200 μL) were transferred to tubes and the reaction was terminated by the addition of an equal volume of ice-cold methanol, which resulted in immediate cell lysis. Then, samples were centrifuged at 3,000 rpm for 10 min and the supernatants transferred to an opaque 96-well plate and the fluorescence was read using Gene5 2.01 Software in Synergy H1 Hybrid Multi-Mode Microplate Reader (BioTek, AHSI, Milan Italy) with excitation and emission at 530 and 590 nm, respectively. Standard curves for resorufin formation were also performed. Data were normalized to total protein content, which was determined using the Bradford reagent according to the manufacturer’s recommendations (Sigma-Aldrich, Milan, Italy).

### Proliferation assay

Cells (1 × 10^5^) were seeded in 24-well plates in regular growth medium, washed once they had attached and then incubated in medium containing 2.5% charcoal-stripped FBS, transfected for 24 h and then exposed to treatments. Transfection were renewed every 2 days and treatments every days. Cells were counted on day 5 using the Countess Automated Cell Counter, as recommended by the manufacturer’s protocol (Life Technologies, Milan, Italy).

### *In vivo* studies

Female 45-day-old athymic nude mice (nu/nu Swiss; Envigo Laboratories) were maintained in a sterile environment. At day 0, exponentially growing MDA-MB-231 cells (2.5 × 10^6^ per mouse) were inoculated in mammary fat pad in 0.1 mL of Matrigel (Cultrex; Trevigen Inc.). When the tumors reached average ∼0.15 cm^3^ (i.e., in about 1 week), mice were randomized and divided into four groups, according to treatments administered by intramuscular (G-1) and/or subcutaneous (TMS) injection for 21 days. The first group of mice (*n* = 7) was treated daily with vehicle (0.9% NaCl with 0.1% albumin and 0.1% Tween-20; Sigma-Aldrich), the second group of mice (*n* = 7) was treated daily with G-1 (0.5 mg/kg/die), the third group of mice (*n* = 7) was treated daily with TMS (0.3 mg/kg/die), and the fourth group of mice (*n* = 7) was treated daily with G-1 in combination with TMS (the concentrations were similar to those described above). G-1 and TMS were dissolved in DMSO at 1 mg/mL. MDA-MB-231 xenograft tumor growth was evaluated twice a week by caliper measurements, along two orthogonal axes: length (L) and width (W). Tumor volumes (in cubic centimeters) were estimated by the following formula: TV = L × (W2)/2. After 21 days of treatment, the animals were killed following the standard protocols and tumors were dissected from the neighboring connective tissue. Specimens of tumors were frozen in nitrogen and stored at –80°C; the remaining tumor tissues of each sample were fixed in 4% paraformaldehyde and embedded in paraffin for the histologic analyses. Animal experiments were conducted according to Italian law (D.L. 26/2014), the Guide for Care and Use of Laboratory Animals published by the US National Institutes of Health (2011), and the Directive 2010/63/EU of the European Parliament on the protection of animals used for Scientific research. The animal research project was approved by the Italian Ministry of Health, Rome (authorization n. 199/2015-PR).

### Immunohistochemistry

Paraffin embedded sections, 5 μm thick, were mounted on slides precoated with poly-lysine, and then they were deparaffinized and dehydrated (7–8 serial sections). Immunohistochemical experiments were performed after heat-mediated antigen retrieval. Hydrogen peroxide (3% in distilled water) was used, for 30 min, to inhibit endogenous peroxidase activity while normal goat serum (10%) was utilized, for 30 min, to block the non-specific binding sites. Immunodetection was carried out using anti-Ki67 and cyclin D1 (1:100) (DAKO, Denmark), cyclin E (1:200) (Bethyl Laboratories, Texas, USA) and cyclin A (1:50) (Abcam, DBA) primary antibodies at 4°C overnight. Then, a universal biotinylated IgG was applied (1:600) for 1 hour at RT, followed by ABC/HRP. Immunoreactivity was visualized by using DAB. The negative controls were made with DAKO mouse IGg1 (cod.X0931) for Ki67, DAKO immunoglobulin fraction (cod.X0936) for cyclin D1 at the same concentration of primary antibodies, rabbit serum at 5% for cyclin E and cyclin A. Sections were also counterstained with haematoxylin. Six-seven serial sections were processed for each sample from two independent operators.

### Imaging

Tissue samples were visualized using an OLYMPUS BX41 microscope (Olympus Europa, Germany) and the images were taken with CSV1.14 software using a CAM XC-30 for images acquisition.

### Statistical analysis

Statistical analysis was performed using ANOVA followed by Newman-Keuls’ testing to determine differences in means. Statistical comparisons for *in vivo* studies were made using the Wilcoxon–Mann–Whitney test. *P* < 0.05 was considered statistically significant.

## SUPPLEMENTARY MATERIALS FIGURES



## Abbreviations

4OHE2: 4-Hydroxyestradiol
CAFs: Cancer-associated fibroblasts
CYP1B1: Cytochrome P450, family 1, subfamily B, polypeptide 1
E2: 17β-estradiol
EGFR: Epidermal growth factor receptor
ERE: estrogen responsive element
EROD: Ethoxyresorufin-O-deethylase activity assay
ERα: Estrogen receptor α
GPER: G protein estrogen receptor
MAPK: Mitogen-activated protein kinase
met-CAFs: CAFs derived from a cutaneous metastasis of an invasive mammary ductal carcinoma
TIS: Transcription initiation site
TMS: 1-[2,(3,5-Dimethoxyphenyl)ethenyl]-2,4-dimethoxybenzene
