# Supplementary material for: GPER is involved in the regulation of the estrogen-metabolizing CYP1B1 enzyme in breast cancer
Source: Oncotarget. 2017 Nov 20;8(63):106608–24. doi: 10.18632/oncotarget.22541 (PMC5739760; doi:10.18632/oncotarget.22541)
Supplement: Supplementary file 1 [file oncotarget-08-106608-s001.pdf]

GPER is involved in the regulation of the estrogen-metabolizing CYP1B1 enzyme in breast cancer

SUPPLEMENTARY MATERIALS

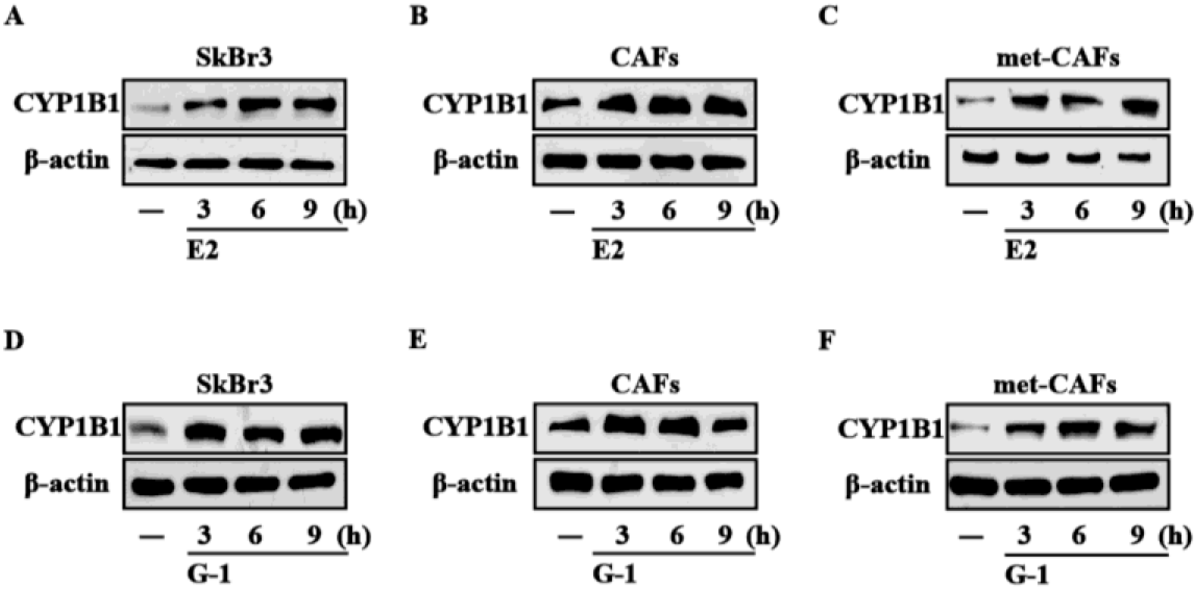

Supplementary Figure 1: GPER mediates CYP1B1 protein induction by E2 and G-1 in SkBr3 cells, CAFs and met-CAFs. Evaluation of CYP1B1 protein levels in SkBr3 cells, CAFs and met-CAFs treated with 10 nM E2 (**A**–**C**) and 100 nM G-1 (**D**–**F**), as indicated. β-actin serves as a loading control. Results shown are representative of at least two independent experiments.

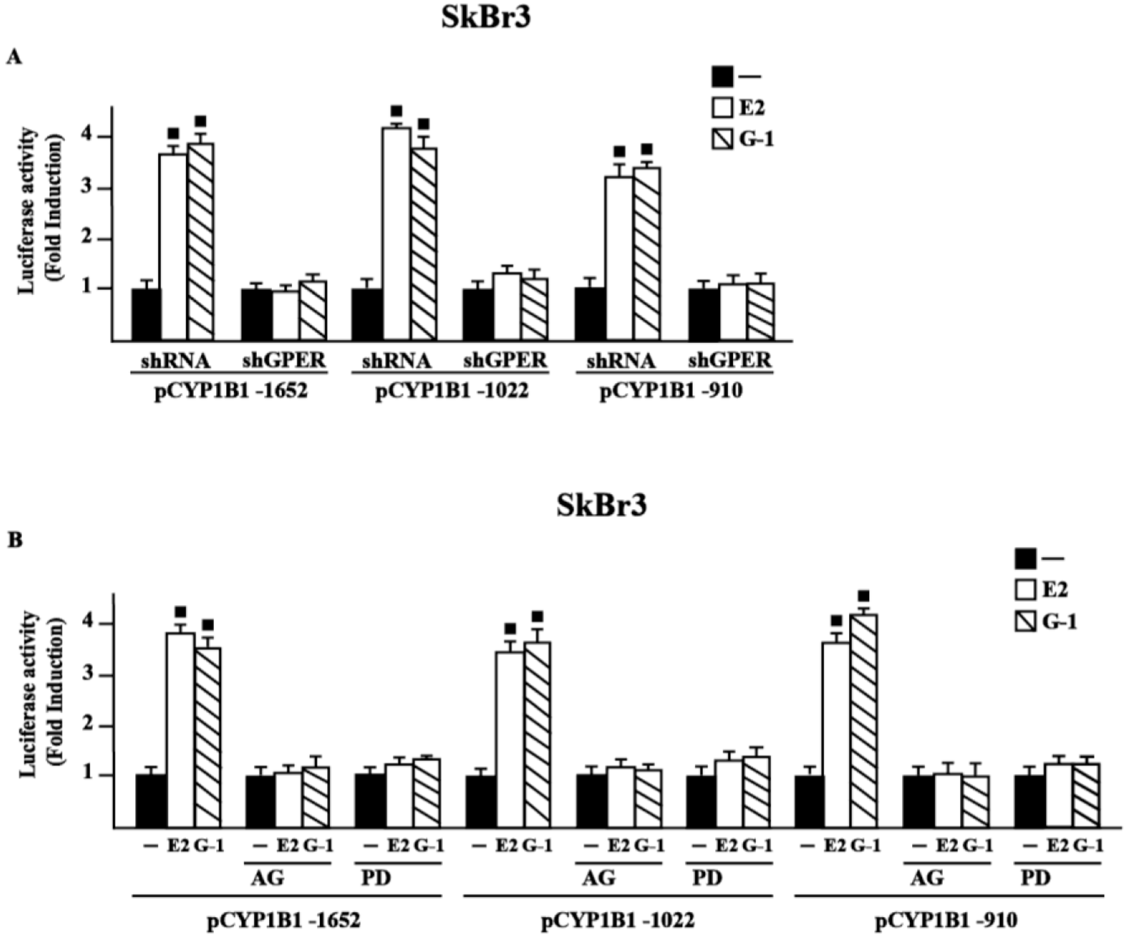

Supplementary Figure 2: GPER mediates the transactivation of CYP1B1 promoter constructs induced by E2 and G-1 in SkBr3 cells. (**A**) SkBr3 cells were transfected for 24 h with shRNA or shGPER, then transfected for 8 h with CYP1B1 promoter constructs and thereafter treated for 18 h with vehicle (−), 10 nM E2 and 100 nM G-1, as indicated. (**B**) Luciferase activities of CYP1B1 promoter constructs in SkBr3 cells treated for 18 h with vehicle, 10 nM E2, 100 nM G-1 alone or in combination with 10 µM EGFR inhibitor AG1478 (AG) and 10 µM MEK inhibitor PD98059 (PD), as indicated. The luciferase activities were normalized to the internal transfection control and values of cells receiving vehicle were set as 1-fold induction upon which the activities induced by treatments were calculated. Each column represents the mean ± SD for three independent experiments, each performed in triplicate. (■) indicates $P < 0.05$ for cells receiving treatments versus vehicle.

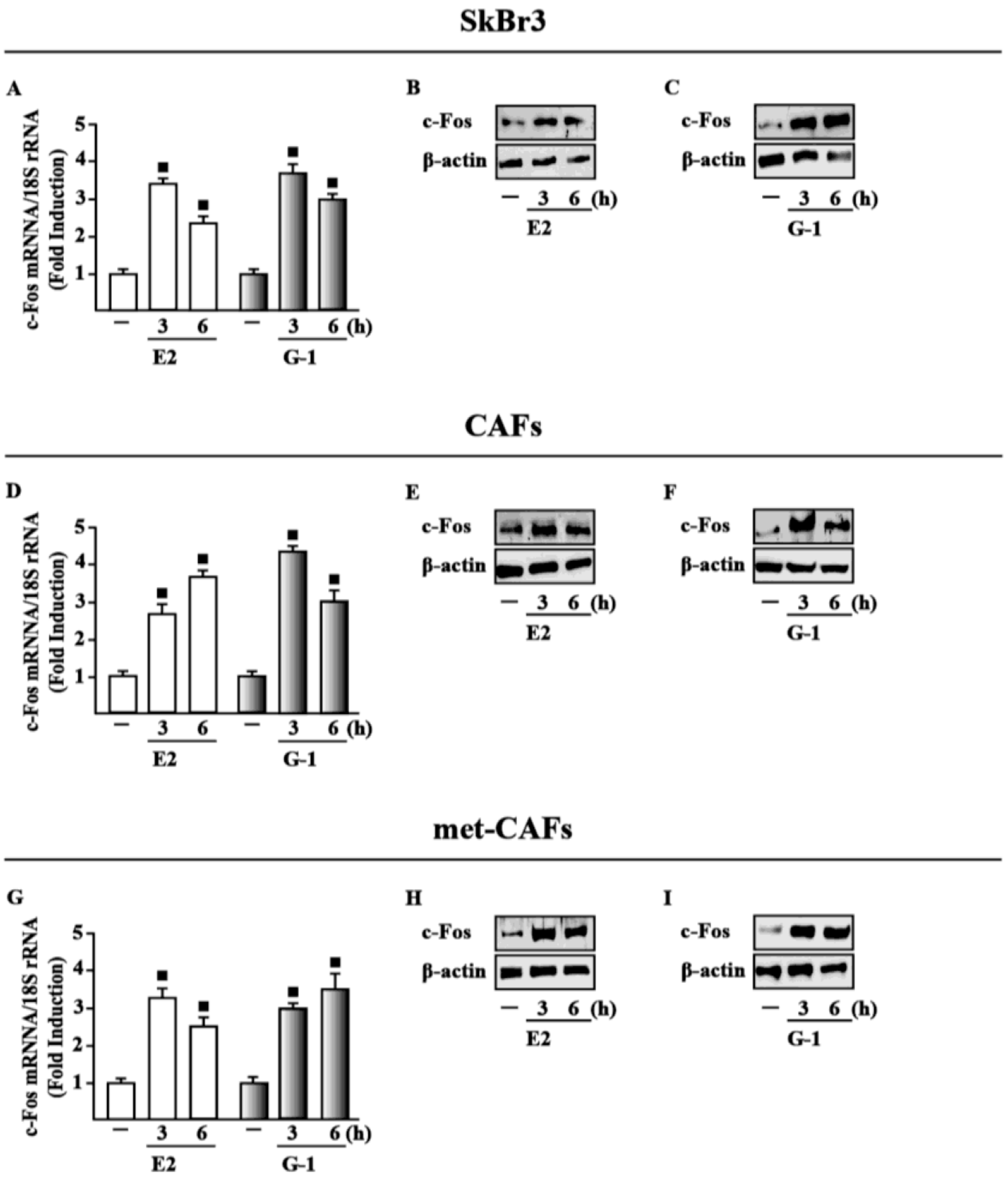

Supplementary Figure 3: E2 and G-1 stimulate c-Fos expression in SkBr3 cells, CAFs and met-CAFs. c-Fos mRNA expression by real-time PCR in SkBr3 cells (**A**), CAFs (**D**) and met-CAFs (**G**) treated with vehicle (−), 10 nM E2 and 100 nM G-1, as indicated. Data obtained in three independent experiments performed in triplicate were normalized to 18S expression and shown as fold changes of CYP1B1 expression upon E2 and G-1 treatments respect to cells treated with vehicle. (■) $P < 0.05$ for cells receiving treatments versus vehicle. c-Fos protein levels in SkBr3 cells (**B**–**C**), CAFs (**E**–**F**), met-CAFs (**H**–**I**) treated with vehicle, 10 nM E2 and 100 nM G-1, as indicated. β-actin serves as a loading control. Results shown are representative of at least two independent experiments.
